# Global Single-Cell Sequencing Landscape of Adipose Tissue of Different Anatomical Site Origin in Humans

**DOI:** 10.1155/2023/8282961

**Published:** 2023-05-08

**Authors:** Shixing Gu, Zhenyu Gong, Shuncai Liu, Guohao Lu, Yu Ling, Yanlin Wei, Ting Li, Ronghe Gu, Yongxian Rong, Junjun Li, Hongmian Li

**Affiliations:** ^1^Department of Plastic and Aesthetic Surgery, The Affiliated Hospital of Youjiang Medical University for Nationalities, Baise, 533022 Guangxi, China; ^2^Department of Burn, Plastic and Aesthetic Surgery, Affiliated Hospital of Guilin Medical University, Guilin, 541001 Guangxi, China; ^3^Department of Emergency, The People's Hospital of Guangxi Zhuang Autonomous Region, Nanning, Guangxi 53002, China; ^4^Department of Basic Science, YuanDong International Academy of Life Sciences, Hong Kong 999077, China; ^5^Department of Orthopedics, The Fifth Affiliated Hospital of Guangxi Medical University & The First People's Hospital of Nanning, Nanning, 53002 Guangxi, China; ^6^Department of Burn, Plastic and Aesthetic Surgery, The Guiping People's Hospital, Guigping, 537200, China; ^7^Department of Pediatrics, The People's Hospital of Guangxi Zhuang Autonomous Region & Institute of Hospital Management and Medical Prevention Collaborative Innovation, Guangxi Academy of Medical Sciences, Nanning, 530021 Guangxi, China; ^8^Department of Plastic and Reconstructive Surgery, The People's Hospital of Guangxi Zhuang Autonomous Region & Research Center of Medical Sciences, Guangxi Academy of Medical Sciences, Nanning, 530021 Guangxi, China

## Abstract

Chronic refractory wounds (CRW) are one of the most serious clinical challenges for surgeons to address. Stromal vascular fraction gels (SVFG), including human adipose stem cells (hASCs), have excellent vascular regenerative and tissue repair properties. Here, we combined single-cell RNA sequencing (scRNA-seq) of leg subcutaneous adipose tissue samples with scRNA-seq data from abdominal subcutaneous adipose tissue, leg subcutaneous adipose tissue, and visceral adipose tissue samples from public databases. The results showed specific differences in cellular levels in adipose tissue from different anatomical site sources. We identified cells including CD4^+^ T cells, hASCs, adipocyte (APC), epithelial (Ep) cells, and preadipocyte. In particular, the dynamics between groups of hASCs, epithelial cells, APCs, and precursor cells in adipose tissue of different anatomical site origins were more significant. Furthermore, our analysis reveals alterations at the cellular level and molecular level, as well as the biological signaling pathways involved in these subpopulations of cells with specific alterations. In particular, certain subpopulations of hASCs have higher cell stemness, which may be related to lipogenic differentiation capacity and may be beneficial in promoting CRW treatment and healing. In general, our study captures a human single-cell transcriptome profile across adipose depots, the cell type identification and analysis of which may help dissect the function and role of cells with specific alterations present in adipose tissue and may provide new ideas and approaches for the treatment of CRW in the clinical setting.

## 1. Introduction

Chronic refractory wounds (CRW), commonly known as chronic ulcers, are defined by the International Society for Wound Healing as wounds that are unable to achieve anatomical and functional integrity through a normal orderly and timely repair process [1]. These wounds are all characterized by difficulty in healing, long duration of disease, and inability to achieve the desired results with drug exchange therapy alone or with drugs in combination with traditional surgery, which often has a large negative impact on the patient's physiology and psychology and causes a heavy economic burden to the patient's family [2].

Adipose tissue is a highly plastic organ that plays an important role in the energy metabolism of the entire body [3]. Adipocytes are capable of storing chemical energy in lipid droplets, thus protecting other tissues from the toxic effects of lipid deposits, and the thermogenic activity of adipocytes allows them to protect themselves and other tissues from lipid overaccumulation [4]. In the case of chronic overnutrition, which would lead to obesity, adipose tissue expands, mainly by increasing the size of adipocytes (enlargement) and/or the number of adipocytes (hyperplasia) [5], and the hypertrophy of adipocytes leads to fibrosis and inflammation, which in turn leads to metabolic disturbances [6]. Human adipose tissue is an important source of adult stem cells because of its abundant storage, easy accessibility, low damage to the body during extraction, lack of rejection in autologous transplantation, and good histocompatibility. Stromal vascular fraction gel (SVFG) is a heterogeneous cell population that includes human adipose stem cells (hASCs) and preadipocytes, among others [7]. Numerous clinical experiments have confirmed that SVFG has extremely strong vascular regenerative and tissue repair properties, attributed on the one hand to different cells with different functions, such as hASCs as an adult stem cell with multidirectional differentiation potential, lymphocytes inducing immune tolerance to reduce the immune response in patients, and endothelial/progenitor cells as an important cell population for blood vessel formation [7, 8]. On the other hand, different cells of SVFG secrete a large number of cytokines that can act in coordination with each other, such as vascular endothelial growth factor (VEGF) produced by hASCs that contributes to the migration of endothelial progenitor cells and platelet-derived growth factor (PDGF-BB) produced by endothelial progenitor cells that promotes the proliferation and migration of hASCs [9–11]. SVFG has shown good therapeutic effects in various diseases such as skin soft tissue injury, arthritic lesions, androgenetic alopecia, neurodegenerative disorders, and vascular lesions, making it promising for a wide range of clinical applications [12–16]. On the other hand, new adipocytes differentiated from progenitor cells are essential to maintain the proper functioning of adipose tissue and prevent metabolic disorders [17]. In addition, some stromal cells, such as epithelial cells and fibroblasts, have been shown to play an important role in curing CRW [18–20]. Unfortunately, the exact cell types, cell kinetics, and mechanisms controlling adipocyte growth are not well understood.

However, despite extensive work in characterizing the various cell subpopulations in adipose tissue [21–23], the complete cytology of human adipose tissue has not been explored and understood. Due to the multifactorial nature of adipose tissue function, a thorough understanding of the cell types involved and their specific gene expression patterns is essential. Currently, cellular heterogeneity and functional status can be detected at the single-cell level with high reproducibility and sensitivity using single-cell transcriptome technology [24]. To date, the heterogeneity and developmental interrelationships between adipose tissues of different origins are not fully understood.

We analyzed single-cell RNA sequencing (scRNA-seq) data from leg subcutaneous adipose tissue samples in combination with scRNA-seq of subcutaneous adipose tissue (SAT), leg subcutaneous adipose tissue, and visceral adipose tissue (VAT) samples from public databases, and our results characterize alterations at the cellular level, molecular level, and biological signaling pathways involved in these subpopulations of cells with specific alterations.

## 2. Methods

### 2.1. Data Source for Human Adipose Tissue Samples

Subcutaneous adipose tissue samples from the legs were obtained from a patient who was elderly and had a combination of chronic refractory wounds. This study was approved by the Ethics Committee of the Fifth Affiliated Hospital of Guangxi Medical University (No.2019-107-01). All procedures involving human participants complied with the ethical standards of the research committee and its ethical standards. Informed consent was obtained from the participants for all study procedures and sequencing protocols.

In addition, the GSE129363 dataset from the GPL16791 platform was acquired based on Gene Expression Omnibus (GEO, https://www.ncbi.nlm.nih.gov/geo/), including scRNA-seq data from 1 abdominal SAT sample from 1 human female. The GSE129363 dataset based on the GPL20301 platform includes scRNA-seq data of 25 adipose samples from 14 individuals who underwent bariatric surgery, of which 12 were VAT samples and 13 were SAT samples. In addition, scRNA-seq data of 3 subcutaneous leg adipose tissue samples from 3 healthy female donors were obtained from the Sequence Read Archive (SRA) with accession number SRP148833. For scRNA-seq in public databases, samples that did not match this study were excluded.

### 2.2. Single-Cell Transcriptome Sequencing

Separation of SVF from adipose tissue using mechanical procedures. Sample preparation and cDNA library construction were performed as described in the 10× Genomics Single Cell 3′ v3.1 kit user guide. Briefly, droplets of latex gel beads (GEMs) with cells absorbed were obtained by microfluidic techniques. The GEMs were subsequently broken, recovered, and enriched for cDNA by PCR amplification to complete the library construction of cDNA. The cDNA product and library concentration were detected based on Qubit 4.0 fluorescence quantification instrument, and the insert size of the cDNA library was detected by Qseq400 Bioanalyzer to guarantee qualified insert size, single peak type, no spurious peak, no junction, and no primer dimer. Finally, the sample libraries were sequenced using the Novaseq 6000 instrument of the Illumina platform. After the identification of Casava bases, the obtained raw image files are converted to sequence files and stored in fastq format.

Sequencing data were subsequently compared and quantified using the official 10× Genomics software CellRanger. For all downstream analyses, we required cells with at least 1000 UMIS (indicating the number of captured transcripts) mapping to at least 200 unique genes. Also to ensure cell quality, cells were filtered for this project to have gene expression numbers > 500 and mitochondrial gene content of 20% or more.

### 2.3. Clustering of Single Cell Data and Construction of Graphs

The IntegrateData function of the Seurat [25] package in R was used to merge single-cell data and annotate clusters using known marker genes that overlap in cluster-specific genes [26]. Differential gene expression analysis between cell clusters was performed using the Wilcoxon rank sum test in the “FindMarkers” function. For Seurat clustering of raw expression data from filtered cells, annotations are generated using SingleR with default parameters. Clustering results were uniformly downscaled and visualized using a uniform manifold approximation and projection (UMAP) for the dimension reduction algorithm [27]. In addition, identified cells are subclustered using the Seurat package to identify marker genes expressed in each cell subcluster using the FindAllMarkers function. Cell subclusters were then classified according to the most abundantly expressed marker genes.

### 2.4. Functional Enrichment Analysis

Gene Ontology (GO) and Kyoto Encyclopedia of Genes and Genomes (KEGG) enrichment analyses were applied to determine the potential function of molecular pathways occurring in each cell subpopulation. clusterProfiler package in R was used for enrichment analysis [28]. Pathways enriched by marker genes were considered significant when *P* < 0.05.

### 2.5. Pseudotime Analysis

During the growth and development of life, cells are constantly transitioning from one functional state to another. During the transition process, it undergoes transcriptional recombination. Thus, the use of pseudotime analysis allows for the sequencing of single cell trajectories based on the expression pattern of the cells. We performed a proposed temporal trajectory analysis of cell subpopulations using the R package Monocle3 [29], which shows the pseudotime changes of cell subpopulations by UMAP plots.

### 2.6. Gene Regulatory Network

Using single-cell regulatory network inference and clustering (SCENIC) to infer gene regulatory networks and identify cellular states based on single-cell expression profiles provides an important biological perspective on the mechanisms driving cellular heterogeneity. To identify internal transcriptional regulatory drivers in adipose tissue of different anatomical site origins, we analyzed and reconstructed gene regulatory networks centered on TFs using the python module tool pySCENIC [30, 31].

### 2.7. Data Analysis and Statistics

All bioinformatic analyses in this study were performed based on the Bioinforcloud platform (http://www.bioinforcloud.org.cn).

## 3. Results

### 3.1. Treatment of Stromal Vascular Fraction Gel (SVFG) in an Elderly Patient with a Combination of Chronic Refractory Trauma

The patient, an 86-year-old female with cerebral infarction and cognitive dysfunction, had multiple pressure ulcers combined with aspergillosis (Figure S1A), which was treated with glucocorticoids for a long time but did not heal. In addition, the main clinical features of this patient were anemia, hypoproteinemia, malnutrition, and extreme wasting. He was then transferred to the Neurosurgery Department of the Fifth Affiliated Hospital of Guangxi Medical University on February 6, 2018. After admission, by combining SVFG adjuvant therapy with conventional myocutaneous flap grafting (Figure S1B), the patient's wound healing time was significantly shortened, healing speed was accelerated, and healing quality was significantly improved (Figure S1C-E), and follow-up revealed no recurrence 3 years after treatment.

### 3.2. Global Single-Cell Atlas of Adipose Tissue from Different Anatomical Sites of Human Origin

We obtained one subcutaneous adipose tissue sample from the leg for single-cell transcriptome testing from a patient with advanced age and combined chronic refractory trauma. In addition, we combined scRNA-seq data from sequencing public databases to obtain 29 adipose tissue samples from 18 donors, including 14 SAT, 12 VAT, and 3 subcutaneous leg adipose tissues, to further explore the potential ecological panoply of adipose tissue of different anatomical site origin in humans.

**Figure 1:**
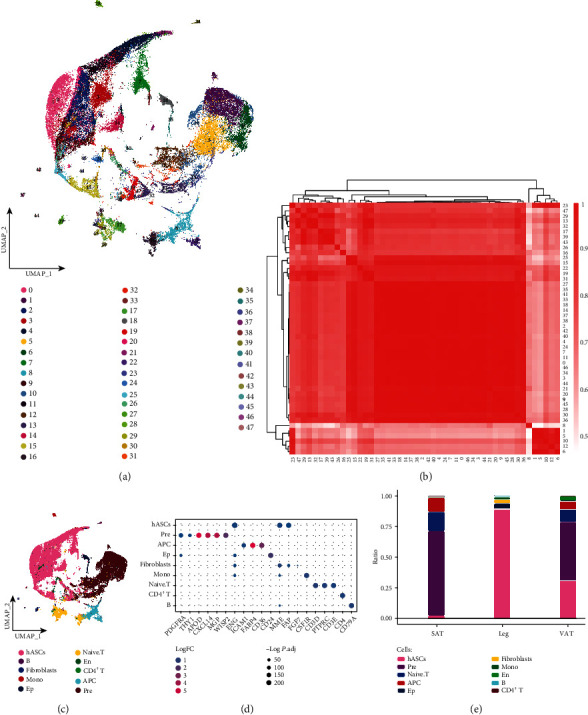
Global single-cell atlas of adipose tissue from different anatomical sources. (a) Single-cell atlas capturing 55,093 cells from 48 cell clusters. (b) Correlation of expression patterns between single cell clusters. (c) Single-cell atlas mapping cell types. (d) Marker genes guiding cell annotation. (e) Differences in cell abundance of adipose tissue of different anatomical site origin in humans.

After standardized data processing and quality control, a total of 55,093 high-quality single-cell transcription profiles were captured and clustered to generate 48 cell clusters (Figures 1(a) and 1(b)), 10 cell types were obtained, including fibroblasts, human adipose stem cells (hASCs), adipocytes (APCs), epithelial cells, and preadipocyte (Pre) (Figure 1(c)). The cell cluster's positively expressed markers were consistent with the gene signatures published by recent scRNA-seq and laboratory studies, among others [3, 32, 33], consistent with the phenotypic characteristics of the corresponding cells (Figure 1(d)). Further comparison of differences in cell composition between adipose tissues of different anatomical origin in humans revealed a significant abundance of hASCs in subcutaneous adipose tissue of the legs, and a significant abundance of APC and preadipocyte in both SAT and VAT was observed (Figure 1(e)). In summary, we initially constructed a dynamic single-cell ecological global landscape of adipose tissue of different anatomical site origins in humans by single-cell histology, and we found that different cell types have ecological alterations in adipose tissue of different anatomical site origins.

### 3.3. Associated Adipocytes Subpopulations in Adipose Tissue of Different Anatomical Origin

**Figure 2:**
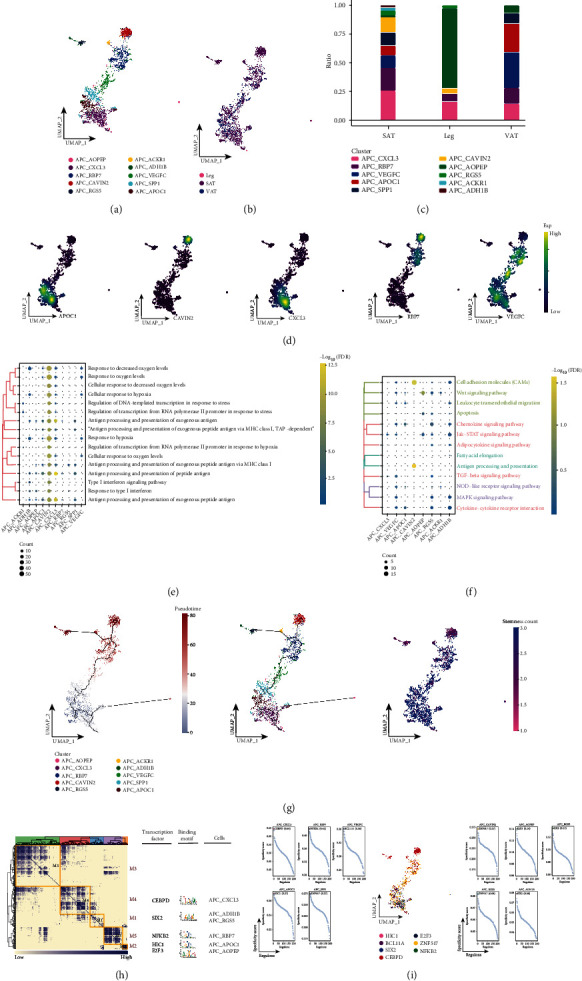
Associated APC subpopulations in adipose tissue of different anatomical origin. (a. Single-cell atlas showing APC subpopulations. (b) Single-cell atlas showing APC subpopulations in abdominal subcutaneous adipose tissue-leg adipose tissue-visceral adipose tissue. (c) Differential abundance of APC subpopulations in abdominal subcutaneous adipose tissue-leg adipose tissue-visceral adipose tissue. (d) Series of single-cell atlases showing markers for specific cell subpopulations. (e) Biological processes are significantly activated by APC subpopulations. (f) Signaling pathways are significantly activated by APC subpopulations. (g) Pseudotime analysis demonstrating pseudotime values (left) and pseudotime trajectories (middle), and cell stemness (right) of APC subpopulations. (h) Coexpression modules of transcription factors in APC. Identification of regulator modules based on the connection specificity index (CSI) matrix of regulators (left). Representative transcription factors and their binding patterns in the modules (middle:). Cell subpopulations where transcription factors are located (right). (i) Transcription factors that have regulatory effects on APC subpopulations.

Based on cellular ecological mapping at single-cell resolution, we deeply explored the associated APC subpopulations in adipose tissues of different anatomical origins and identified a total of 10 subpopulations of APC (Figure 2(a)) and found that these subpopulations were heterogeneous among different subpopulations, in particular, these subpopulations were largely present in SAT and VAT groups, which was consistent with the results of Figure 1(e) (Figure 2(b)). Further exploring the variation in the abundance of APC subpopulations, we found that APC_CXCL3 had the highest proportion in the subcutaneous adipose tissue of the leg, APC_RBP7 and APC_CAVIN2 in SAT, APC_VEGFC in VAT, and APC_APOC1 only in SAT and VAT (Figure 2(c)), while the specific markers of these specific APC subpopulations were mapped in the single-cell atlas (Figure 2(d)). By enrichment analysis, these specifically altered APC subpopulations were found to be significantly involved in the biological processes of several oxidative responses (Figure 2(e)), with significant enrichment in the Wnt signaling pathway, antigen processing and presentation, cell adhesion molecules (CAMs), and cytokine receptor interaction pathways (Figure 2(f)). These APC subpopulations underwent a continuous developmental process in which APC_CXCL3 was located at the beginning of development with high cell stemness, and APC_CAVIN2 and APC_RBP7 were located at the end of development with the lowest cell stemness (Figure 2(g)). Subsequent GRN analysis showed that this subpopulation of genes was organized into five modules (Figure 2(h)), each regulated by different transcription factors (TFs) specific to the APC subpopulation (Figure 2(i)). These results reflect changes in the relevant APC subpopulations in adipose tissue of different anatomical origins, as well as the signaling pathways and pseudotime changes involved in tissue-specific APC subpopulations.

### 3.4. Associate Edepithelial Cell Subpopulations in Adipose Tissue of Different Anatomical Origin

**Figure 3:**
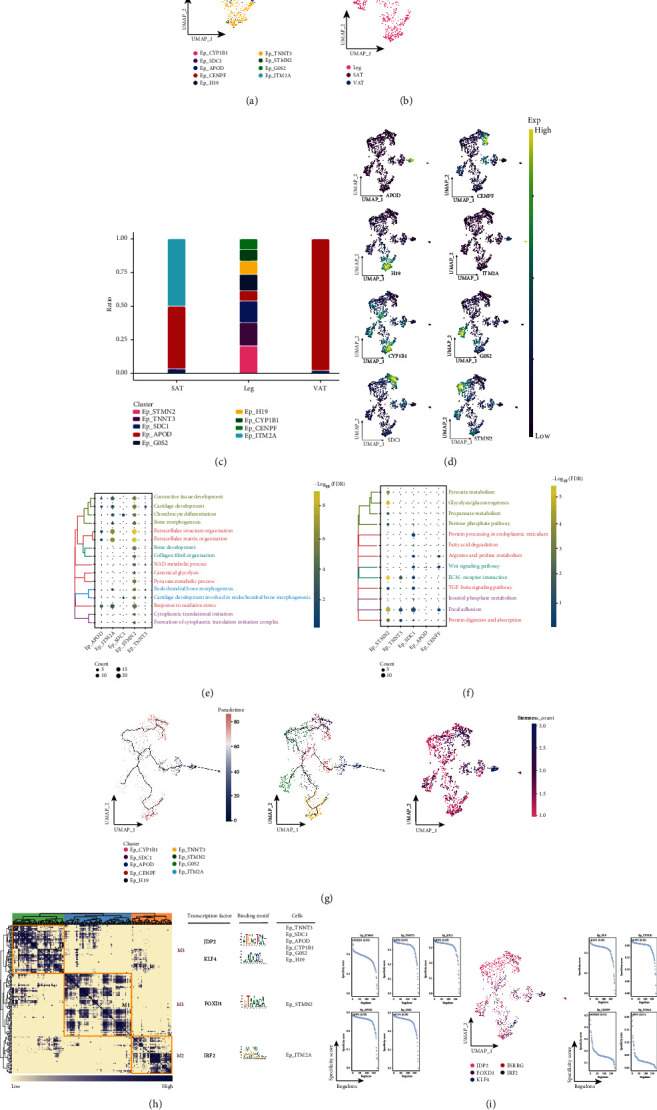
Associated Ep cell subpopulations in adipose tissue of different anatomical origins. (a) Single-cell atlas showing Ep cell subpopulations. (b) Single-cell atlas showing EP cell subpopulations in abdominal subcutaneous adipose tissue-leg adipose tissue-visceral adipose tissue. (c) Differential abundance of Ep cell subpopulations in abdominal subcutaneous adipose tissue-leg adipose tissue-visceral adipose tissue. (d) Series of single-cell atlases showing markers for specific cell subpopulations. (e) Biological processes are significantly activated by Ep cell subpopulations. (f) Signaling pathways significantly activated by Ep cell subpopulations. (g) Pseudotime analysis demonstrating pseudotime values (left), pseudotime trajectories (middle), and cell stemness (right) of Ep cell subpopulations. (h) Coexpression modules of transcription factors in Ep cells. Identification of regulator modules based on the connection specificity index (CSI) matrix of regulators (left). Representative transcription factors and their binding patterns in the modules (middle). Cell subpopulations in which transcription factors are located (right). (i) Transcription factors that have regulatory effects on Ep cell subpopulations.

The process of wound healing involves the migration and proliferation of epithelial cells, which are stimulated by epidermal growth factor, transforming growth factor, and transforming growth factor alpha to cover new tissue [34]. For Ep subpopulations, a total of nine subpopulations of Ep were identified (Figure 3(a)), and most of these subpopulations were found to be present in the subcutaneous adipose tissue of the legs (Figure 3(b)). In addition, Ep_APOD was significantly abundant in SAT and VAT, while Ep_STMN2, Ep_TNNT3, Ep_SDC1, Ep_G0S2, Ep_H19, Ep_CYP1B1, Ep_CENPF, and Ep_ITM2A had the highest proportions in subcutaneous adipose tissue of the legs, and Ep_TNNT3 and Ep_H19 had the highest proportions in SAT, indicating a high heterogeneity of Ep subpopulations in subcutaneous adipose tissue of the legs (Figure 3(c)), while specific markers for these specific Ep subpopulations were mapped in the single cell atlas (Figure 3(d)). By enrichment analysis, these specifically altered Ep subpopulations were found to be significantly involved in skeletal development as well as in biological processes related to oxidative stress (Figure 3(e)), and significantly enriched in metabolism-related pathways (Figure 3(f)). In addition, the pseudotime analysis revealed that Ep_TNNT3 is located at the beginning of development and has a higher cell stemness, and Ep_TNNT3, Ep_SDC1, and Ep_CENPF are located at the end of development and have a lower cell stemness (Figure 3(g)). Subsequent GRN analysis showed that this subgroup of genes was organized into three modules (Figure 3(h)), each regulated by different TFs (Figure 3(i)). These results reflect changes in the relevant Ep subpopulations in adipose tissue of different anatomical origins, as well as the signaling pathways and pseudotime changes involved in tissue-specific Ep subpopulations.

### 3.5. Associated Fibroblast Subpopulations in Adipose Tissue of Different Anatomical Origin

**Figure 4:**
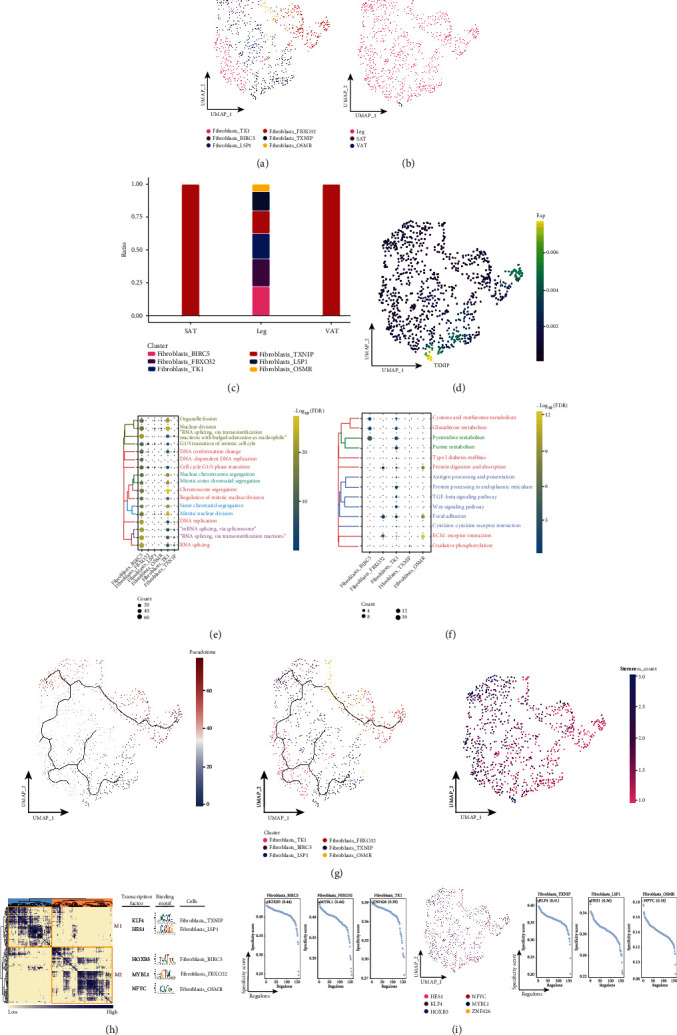
Associated fibroblast subpopulations in adipose tissue of different anatomical origins. (a) Single-cell atlas showing fibroblast subpopulations. (b) Single-cell atlas showing fibroblast subpopulations in abdominal subcutaneous adipose tissue-leg adipose tissue-visceral adipose tissue. (c) Differential abundance of fibroblast subpopulations in abdominal subcutaneous adipose tissue-leg adipose tissue-visceral adipose tissue. (d) Series of single-cell atlases showing markers for specific cell subpopulations. (e) Biological processes are significantly activated by fibroblast subpopulations. (f) Signaling pathways are significantly activated by fibroblast subpopulations. (g) Pseudotime analysis demonstrating pseudotime values (left), pseudotime trajectories (middle), and cell stemness (right) for fibroblast subpopulations. (h) Coexpression modules of transcription factors in fibroblasts. Identification of regulator modules based on the connection specificity index (CSI) matrix of regulators (left). Representative transcription factors and their binding patterns in the modules (middle). Cell subpopulations where transcription factors are located (right). (i) Transcription factors that have regulatory effects on fibroblast subpopulations.

The main cells involved in the proliferative phase of the wound-healing process consist of fibroblasts [34]. For fibroblast subpopulations, a total of six fibroblast subpopulations were identified (Figure 4(a)) and were found to be largely derived from subcutaneous adipose tissue of the leg (Figure 4(b)). In addition, we found that fibroblast_TXNIP was significantly abundant in SAT and VAT, which caught our attention (Figure 4(c)), and specifically expressed TXNIP (Figure 4(d)). Enrichment analysis revealed that the fibroblast subpopulation of fibroblast_TXNIP was significantly involved in cell cycle-related biological processes (Figure 4(e)) and enriched in oxidative phosphorylation, while other subpopulations were involved in several metabolism-related pathways (Figure 4(f)). And the pseudotime analysis revealed that fibroblast_TXNIP is located at the beginning of development and has a high cell stemness (Figure 3(g)). Subsequent GRN analysis showed that this subcluster of genes was organized into two modules (Figure 4(h)), which were regulated by different TFs (Figure 4(i)). These results reflect changes in the relevant fibroblast subpopulations in adipose tissue of different anatomical origins, as well as the signaling pathways and pseudotime changes involved in tissue-specific fibroblast subpopulations.

### 3.6. Associated Subpopulations of Human Adipose Stem Cells in Adipose Tissue of Different Anatomical Origin

**Figure 5:**
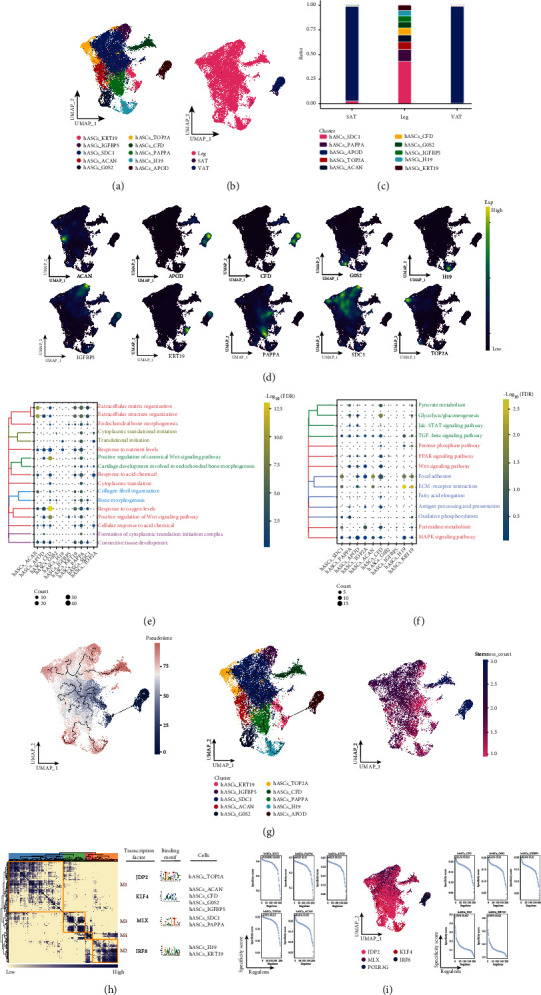
Subpopulations of associated hASCs cells in adipose tissue of different anatomical origins. (a) Single-cell atlas showing hASCs subpopulations. (b) Single-cell atlas showing hASCs subpopulations in abdominal subcutaneous adipose tissue-leg adipose tissue-visceral adipose tissue. (c) Differential abundance of hASCs subpopulations in abdominal subcutaneous adipose tissue-leg adipose tissue-visceral adipose tissue. (d) Series of single-cell atlases showing markers for specific cell subpopulations. (e) Biological processes are significantly activated by hASCs subpopulations. (f) Signaling pathways are significantly activated by hASCs subpopulations. (g) Pseudotime analysis demonstrating pseudotime values (left), pseudotime trajectories (middle), and cell stemness (right) of hASCs subpopulations. (h) Coexpression modules of transcription factors in hASCs. Identification of regulator modules based on the connection specificity index (CSI) matrix of regulators (left). Representative transcription factors and their binding patterns in the modules (middle). cell subpopulations where transcription factors are located (right). (i) Transcription factors that have regulatory effects on hASCs subpopulations.

For hASCs that were significantly abundant in the subcutaneous adipose tissue of the legs, we explored the subpopulations of hASCs in depth based on cellular ecological mapping at single-cell resolution and identified a total of 10 subpopulations of hASCs (Figure 5(a)) and found that these subpopulations were heterogeneous among different subpopulations (Figure 5(b)). Analysis of cell abundance revealed that hASCs_APOD was significantly abundant in SAT and VAT, while hASCs_SDC1, hASCs_PAPPA, hASCs_TOP2A, hASCs_ACAN, hASCs_CFD, hASCs_G0S2, hASCs_IGFBP5, and hASCs_H19 were specifically present in subcutaneous adipose tissue of the legs (Figure 5(c)), while specific markers for these fibroblast subpopulations were mapped in single cell profiles (Figure 5(d)). In addition, these subsets of hASCs are significantly involved in connective tissue development, collagen fibril organization, and biological processes related to bone formation (Figure 5(e)), are enriched in glycolysis/gluconeogenesis, oxidative phosphorylation, and some metabolism-related pathways and are also involved in the Wnt signaling pathway, MAPK signaling pathway, and PPAR signaling pathway, as well as other lipid metabolism-related pathways (Figure 5(f)). And the pseudotime analysis revealed that hASCs_APOD was located at the beginning of development and had a high cell stemness (Figure 5(g)). Subsequent GRN analysis showed that this subgroup of genes was organized into four modules (Figure 5(h)), each regulated by different TFs (Figure 5(i)). Here, our analysis of a subpopulation of associated hASCs in human adipose tissue of different anatomical origins defines a developmental hierarchy of hASCs, capturing that hASCs_APOD has high cell stemness, which we suggest may be associated with adipogenic differentiation capacity.

### 3.7. Associated Preadipocyte Subpopulations in Adipose Tissue of Different Anatomical Origin

**Figure 6:**
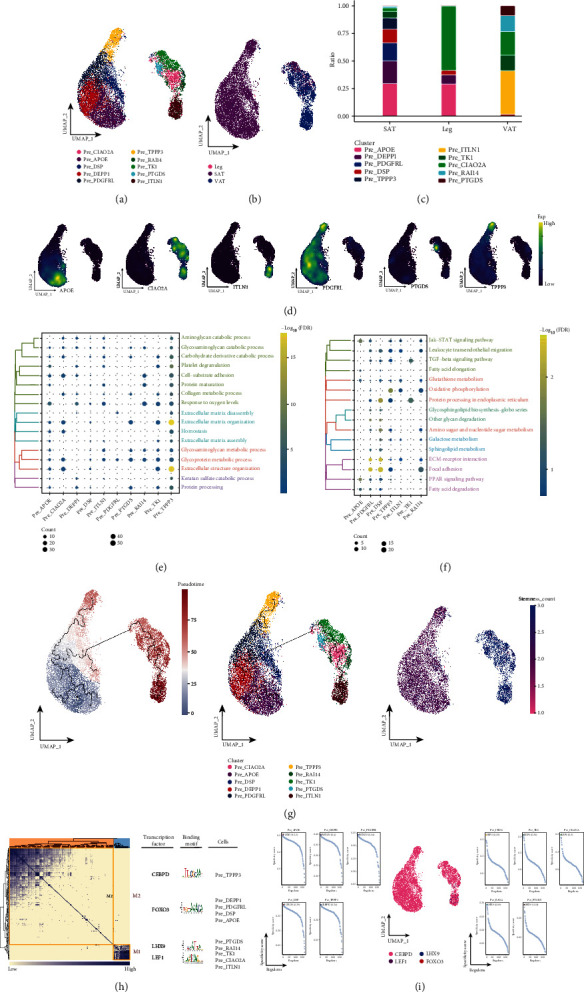
Associated preadipocyte subpopulations in adipose tissue of elderly patients with combined chronic refractory trauma. (a) Single-cell atlas showing preadipocyte subpopulations. (b) Single-cell atlas showing preadipocyte subpopulations in abdominal subcutaneous adipose tissue-leg adipose tissue-visceral adipose tissue. (c) Differential abundance of preadipocyte subpopulations in abdominal subcutaneous adipose tissue-leg adipose tissue-visceral adipose tissue. (d) Series of single-cell atlases showing markers for specific cell subpopulations. (e) Biological processes are significantly activated by preadipocyte subpopulations. (f) Signaling pathways are significantly activated by preadipocyte subpopulations. (g) Pseudotime analysis demonstrating pseudotime values (left), pseudotime trajectories (middle), and cell stemness (right) of preadipocyte subpopulations. (h) Coexpression modules of transcription factors in preadipocyte. Identification of regulator modules based on the connection specificity index (CSI) matrix of regulators (left). Representative transcription factors and their binding patterns in the modules (middle). Cell subpopulations where transcription factors are located (right). (i) Transcription factors that have regulatory effects on preadipocyte subpopulations.

For preadipocyte with significant abundance in SAT and VAT, unsupervised clustering was performed (Figure 6(a)), and 10 preadipocyte subpopulations were captured. The specificity of preadipocyte among different subpopulations was observed by higher resolution (Figures 6(b) and 6(c)), and they have different positive expression gene markers (Figure 6(d)). These preadipocyte subpopulations were enriched for fatty acid degradation, TGF-*β* signaling pathway, PPAR signaling pathway, and some metabolic-related pathways (Figure 6(e)). And at the transcriptome level, these preadipocyte subpopulations undergo a continuous developmental process (Figure 6(g)), where Pre_APOE is at the earliest initiation point in the developmental trajectory (Figure 6(h)). Downstream of it, different gene modules are transcriptionally activated, directing toward different fate choices (Figure 6(i)). Our analysis of relevant preadipocyte subpopulations in human adipose tissue of different anatomical origins revealed that Pre_APOE is at the earliest initiation point in the developmental trajectory and may be a key subpopulation for differentiation into mature adipocytes.

## 4. Discussion

In this study, we provide a comprehensive overview of cell types and subpopulations in human adipose tissue of different anatomical origins at single-cell resolution, providing a single-cell perspective on wound healing after adjuvant treatment with SVFG for CRW. scRNA-seq uniquely characterizes tissue by dissecting cellular heterogeneity at high resolution. This approach has proven to be indispensable to deepen our understanding of biological systems by revealing the complexity of differential variation among cells, as well as the differentiation trajectories of cell subpopulations. A number of studies have used scRNA-seq to characterize the heterogeneity of adipose tissue of different anatomical origins in humans on a temporal scale [3, 22, 35], to obtain gene expression profiles of different cell populations, and to systematically map the cellular-level dynamics of adipose tissue of different anatomical origins in humans. However, the resolution of human adipose tissue ecosystems of different anatomical origins at the single-cell molecular resolution level is still very limited known.

Our initial classification identified 10 separate cell types, including prepipocyte or stem cells, immune cells, and stromal cells, each with unique characteristics, for further in-depth analysis of cell subpopulations. Almost all cells are involved in metabolism-related pathways. It is known that obese individuals, i.e., increased adipose tissue mass, are the main driver of the pathogenesis of metabolic syndrome [36]. Therefore, further exploration of the complexity of adipose tissue biology and its role in metabolism is essential to characterize and corroborate the ecosystem of adipose tissue of different anatomical origin in humans.

SVFG is a group of heterogeneous cells freshly isolated from adipose tissue. It includes smooth muscle cells, preadipocytes, pericytes, and several kinds of pluripotent adult stem cells, such as endothelial progenitor cells and hematopoietic progenitor cells, pericytes, and adipose-derived stem cells (ASCs), among others [37]. Mature adipocytes are derived from ASCs and progenitor cells, while preadipocytes exist in the SVF, but their molecular heterogeneity and functional diversity are still poorly understood [38]. When the local environment is subjected to deleterious signals secondary to metabolic imbalance, hypertrophic adipocyte growth leads to inflammation and fibrosis, as well as the persistence of metabolic dysfunction [3, 38], making the full characterization of APC identity and function critical. Adipose stem cells (ASCs), a type of mesenchymal stem cells (MSCs) [39, 40], have epithelial cell lineage differentiation potential and are attractive candidates for clinical applications to repair or regenerate damaged tissues and organs [41]. Many studies have reported that ASCs can be transdifferentiated into keratinocytes under certain conditions [42, 43]. hASCs have the advantages of having a wide source, easy access strategy, low patient damage, abundant stem cells in adipose tissue, and low immunogenicity [44], suggesting that transdifferentiation of hASCs into keratinocytes could be a promising candidate for promoting a promising strategy for wound healing. Our study captured that hASCs_PAPPA has a high cell stemness, which we believe may correlate with adipogenic differentiation capacity. In addition, we identified preadipocytes specifically present in SAT and VAT that can differentiate into mature adipocytes [3]. Thus, targeting one or more of these cell populations may be beneficial in promoting CRW treatment and healing.

In addition, we identified a series of marker genes with significantly variable cellular subpopulations and, when analyzing their individual gene expression patterns, observed heterogeneity in ecotopic gene expression in individuals in a region-dependent manner. Wound repair is a complex process of interactions between cells, growth factors, and the extracellular matrix [45]. These typical examples further emphasize the complex heterogeneity and cellular plasticity at single-cell resolution. Significantly higher levels of the chemokine CXCL3 were observed [46], and adipose tissue is a biologically active endocrine tissue that serves as an energy store and secretes various proinflammatory cytokines and chemokines [47]. CXCL3 gene expression in human adipocytes mediates a transition from M1 to M2 macrophages that reduces inflammation and leads to faster diabetic wound healing [48]. Previous studies collectively showed that retinol-binding protein 7 (Rbp7) gene expression is adipose tissue-specific among species [49–51]; however, it has not been reported in adipose tissue of human anatomical origin. The lymphatic angiogenic factor VEGF-C has an integral role in metabolic syndrome-associated adipose tissue inflammation, and blockade of VEGF-C improves systemic insulin sensitivity and protects the liver from high-fat diet-induced steatosis, which is associated with reduced adipocyte size and adipose tissue inflammation [52]. In addition, VEGF-C has an important role in promoting wound healing. When the function of endogenous VEGF-C/VEGF-D was blocked by specific inhibitors, wound closure was further delayed [53]. APOC1 has been reported to have an additional role in epidermal lipid synthesis as well as adipose tissue formation [54] due to hyperlipidemia in transgenic mice overexpressing Apoc1. Adipose tissue releases large amounts of bioactive factors called adipokines, many of which are involved in inflammation, glucose homeostasis, and lipid metabolism, such as APOD which is positively associated with the metabolic syndrome. Enhanced ITM2A expression inhibits chondrogenic differentiation of adipose-derived mesenchymal stem cells (55). Overexpression of TXNIP has been shown to induce apoptosis of pancreatic *β*-cells, reduce insulin sensitivity in peripheral tissues such as skeletal muscle and adipose, and decrease energy expenditure (56). In diabetic wound healing, TXNIP maintains the inflammatory response in diabetic wounds (57).

In summary, this study provides a global ecological map of human adipose tissue of different anatomical origins at the single-cell level, including dynamic phenotypes of preadipocyte or stem cells, and stromal cells, focusing on the resolution of parallel evolutionary trajectories and unique features of cell transcription in human adipose tissue of different anatomical origins, highlighting the core drivers that shape adipocytes. Our data can be a valuable resource for constructing a human single-cell transcriptome atlas across adipose depots, the cell type identification and analysis of which may help dissect the function and role of cells with specific alterations present in adipose tissue. In addition, single-cell on-cell insights were provided for patient wound healing. However, this study combines single-cell sequencing and bioinformatics technologies to study adipose tissue of different anatomical origins in humans at the single-cell level, and due to the small amount of sequenced samples, a larger number and diversity of adipose tissue samples should be collected for future studies.

## Data Availability

The datasets analyzed during the current study are available in the GEO repository （https://www.ncbi.nlm.nih.gov/geo/） and Sequence Read Archive (SRA).

## Supplementary Materials

Supplementary Materials Figure S1: stromal vascular fraction gel (SVFG) treatment in elderly patients with combined chronic refractory wounds. A. Chronic refractory wound in case of the patient, wound area: 10 cm × 7 cm. B. Autologous stromal vascular fraction gel (SVFG) injection therapy. C. Myocutaneous flap+SVFG therapy. D. Myocutaneous flap+SVFG therapy two months postoperatively. E. Myocutaneous flap+SVFG therapy 12 months postoperatively.
