# Supplementary material for: Global Single-Cell Sequencing Landscape of Adipose Tissue of Different Anatomical Site Origin in Humans
Source: Stem Cells Int. 2023 May 8;2023:8282961. doi: 10.1155/2023/8282961 (PMC10185425; doi:10.1155/2023/8282961)
Supplement: Supplementary Materials — Figure S1: stromal vascular fraction gel (SVFG) treatment in elderly patients with combined chronic refractory wounds. A. Chronic refractory wound in case of the patient, wound area: 10 cm × 7 cm. B. Autologous stromal vascular fraction gel (SVFG) injection therapy. C. Myocutaneous flap+SVFG therapy. D. Myocutaneous flap+SVFG therapy two months postoperatively. E. Myocutaneous flap+SVFG therapy 12 months postoperatively. [file 8282961.f1.docx]

Supplementary Material

## Supplementary Figures


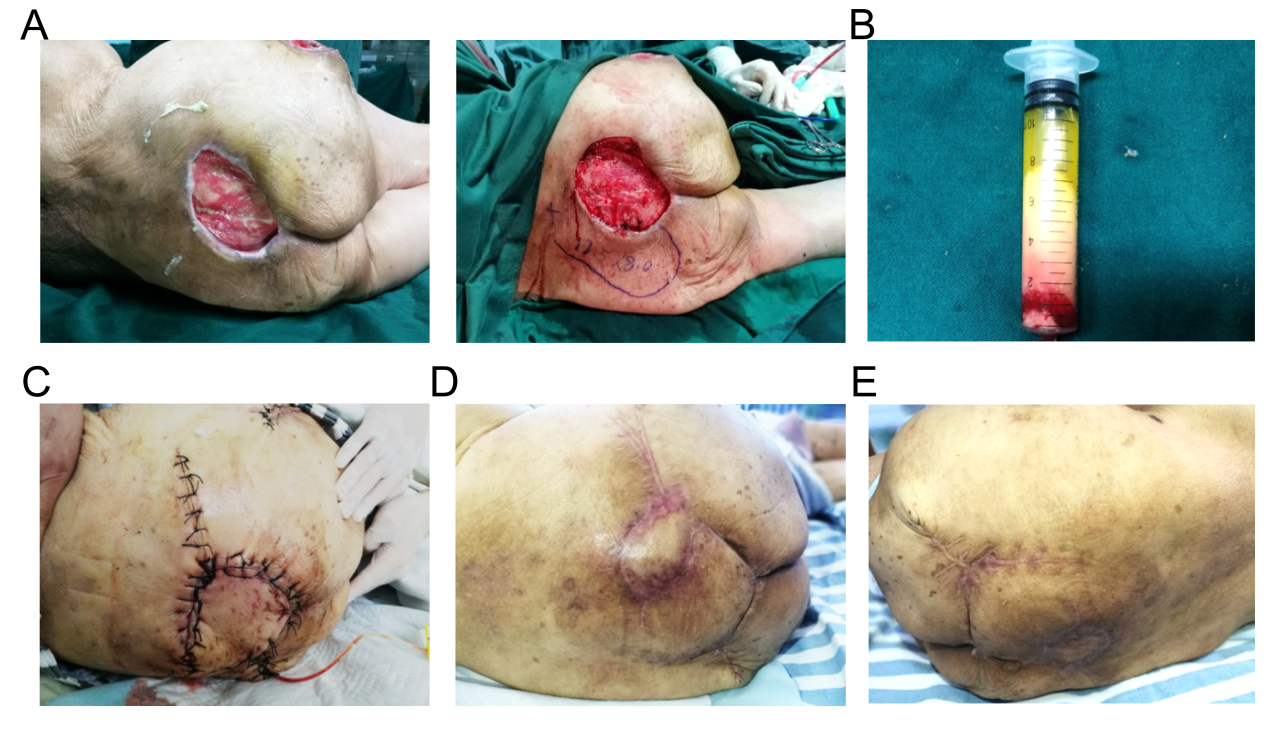


**Figure S1. Stromal vascular fraction gel (SVFG) treatment in elderly patients with combined chronic refractory wounds**

1. Chronic refractory wound in case patient, wound area: 10 cm×7 cm. B. Autologous stromal vascular fraction gel (SVFG) injection therapy. C. Myocutaneous flap +SVFG therapy. D. Myocutaneous flap + SVFG therapy two months postoperatively. E. Myocutaneous flap + SVFG therapy 12 months postoperatively.
